# Screening for the Interaction of Diabetes Mellitus and Other Factors in Tuberculosis

**Published:** 2019-11

**Authors:** Min MU, Jing WU, Jian-Hua WANG, Xin-Rong TAO, Dong HU, Kai LIU, Ke-Hong FANG, Song XUE, Jie SHANG

**Affiliations:** 1. Department of Medical Frontier Experimental Center, School of Medicine, Anhui University of Science and Technology, Anhui, China; 2. Key Laboratory of Industrial Dust Control and Occupational Health, Ministry of Education, Anhui, China; 3. Key Laboratory of Industrial Dust Deep Reduction and Occupational Health and Safety, Anhui Higher Education Institutes, Anhui University of Science and Technology, Anhui, China

## Dear Editor-in-Chief

Tuberculosis has still been identified as a serious public health problem in the world and the single infectious diseases causing the largest number of deaths (1). According to WHO, global TB control in 2009 annual report, 13.7 million people fell ill with TB, 9.27 million became new cases and 1.77 million died from the disease per year (2). Tuberculosis mainly affects the production capacity of people between the age of 15 to 54, which could be a social and economic burden for a country or region (3). Sampling survey results of China's population in 2010 indicated that the number of patients with active tuberculosis is about 4.99 million, of which the number of patients with multi-drug resistant pulmonary tuberculosis is nearly 339 thousand (4). More and more study focuses on clinical treatment of tuberculosis especially the treatment of drug resistance (5). However, many factors such as alcoholism, smoking, nutrition, diabetes can also affect the progress of tuberculosis, at other hands, the cooperation and antagonism among these factors also playa important role to tuberculosis, and it cannot be ignored. At present, less attention is paid to the interactions among these factors.

Therefore, we aimed to identify that interactions association between these factors and tuberculosis. From January 2014, a hospital-based case-control study was conducted in Huainan Oriental Tumor Hospital and Physical Examination Center of Huainan First Hospital, until to November 2017.

Multiple factor conditional logistic regression showed diabetes mellitus has an interactive effect on residence, BMI, and considered as synergism. All tuberculosis cases accounted for interaction between diabetes mellitus and two factors AP 73.4% (95%CI: 0.420–1.048), AP 48.7% (95%CI: 0.176–0.798); RERI 4.762 (−2.463–11.986), 2.999 (0.277–5.721); S 7.554 (1.245–45.827), 2.388 (1.008–5.660). High fat dietary pattern and diabetes mellitus are antagonism, it was statistically significant, AP 55.5% (95%CI: 0.235–0.876); RERI 3.335 (0.334–6.336); S 2.944 (1.004–8.933). Diabetes mellitus and age, passive smoking, medium-intensity exercise, Western food, high VD and Calcium, Traditional Chinese have no statistical significance (Table 1). GMDR was used to analyze the interaction between diabetes and other risk factors, The model with three factors including diabetes, BMI and residence has the highest test balance accuracy (TAB) 0.7073 and Cross-validation consistency(CVC)is 10/10. The *P*-value of the permutation test has statistical significance and the best model. There is interaction between the three-factors (Table 2).

**Table 1:** The additive effect of diabetes and other factor

***Index Diabetes and Age***		***Diabetes and Medium -intensity exercise***		***Diabetes and High VD and Calcium***	
RERI	0.254 (−0.202–0.711)	RERI	2.712 (−1.650–7.075)	RERI	0.648 (−2.427–3.722)
AP	0.163 (−0.418–0.744)	AP	0.607 (0.189–1.025)	AP	0.172 (−0.633–0.978)
S	1.826 (0.018–190.421)	S	4.589 (0.770–27.354)	S	1.307 (0.313–5.462)
Diabetes and Residence		Diabetes and BMI		Diabetes and Traditional Chinese	
RERI	4.762 (−2.463–11.986)	RERI	2.999 (0.277–5.721)	RERI	−1.225 (−7.268–4.819)
AP	0.734 (0.420–1.048)	AP	0.487 (0.176–0.798)	AP	−0.268 (−1.640–1.103)
S	7.554 (1.245–45.827)	S	2.388 (1.008–5.660)	S	0.744 (0.198–2.795)
Diabetes and Passive smoking		Diabetes and Western food		Diabetes and High fat	
RERI	−0.104 (−3.457–3.249)	RERI	−1.040 (−17.825–15.746)	RERI	3.335 (0.334–6.336)
AP	−0.040 (−1.318–1.239)	AP	−0.082 (−1.492–1.328)	AP	0.555 (0.235–0.876)
S	0.940 (0.135–6.549)	S	0.919 (0.223–3.776)	S	2.944 (1.004–8.933)

**Table 2:** GMDR model of High order interaction between diabetes and other factors

***No***	***Model***	***TBA***	**P**	***CVC ***
1	BMI	0.5555	0.1719	6/10
2	BMI, Residence	0.6003	0.0547	8/10
3	Diabetes, BMI, Residence	0.7073	0.0010	10/10
4	Diabetes, BMI, Residence, Western food	0.6829	0.0010	6/10
5	Diabetes, BMI, Residence, Age, Passive smoking	0.7065	0.0010	9/10
6	Diabetes, BMI, Residence, Western food, Medium-intensity exercise, Traditional Chinese	0.6908	0.0010	4/10
7	Diabetes, BMI, Residence, Western food, Medium-intensity exercise, Passive smoking, Traditional Chinese	0.6758	0.0010	4/10
8	Diabetes, BMI, Residence, High fat, Western food, Medium-intensity exercise, Passive smoking,	0.6896	0.0010	8/10
9	Diabetes, BMI, Residence, High VD and Calcium, Traditional Chinese, Medium-intensity exercise, Age, Western food, High fat	0.6776	0.0010	9/10
10	Diabetes, BMI, Residence, High fat, Western food, Age, Medium-intensity exercise, Passive smoking, High VD and Calcium, Traditional Chinese	0.6870	0.0010	10/10

Our study was designed to determine the interaction between diabetes mellitus and other factors of tuberculosis among the population of Huainan city. Given that residence, BMI and diabetes mellitus independently predicted tuberculosis, and synergism was stronger than independent effect. Our results might be meaningful for prevention of tuberculosis. Public health policy should focus on residence, BMI and diabetes mellitus and tuberculosis in China.
